# Mung Bean Protein Supplement Improves Muscular Strength in Healthy, Underactive Vegetarian Adults

**DOI:** 10.3390/nu11102423

**Published:** 2019-10-11

**Authors:** Eric Bartholomae, April Incollingo, Maricarmen Vizcaino, Christopher Wharton, Carol S. Johnston

**Affiliations:** 1 College of Health Solutions, Nutrition Program, Arizona State University, 550 N. 3rd St., Phoenix, AZ 85004, USA; ebartholomae@yahoo.com; 2 College of Health Solutions, Radical Simplicity Lab, Arizona State University, 550 N. 3rd St., Phoenix, AZ 85004, USA; aincolli@asu.edu (A.I.); mvizcain@asu.edu (M.V.); christopher.wharton@asu.edu (C.W.)

**Keywords:** vegetarian, vegan, mung bean protein, lean body mass, muscular strength

## Abstract

Although vegetarian diets are considered generally protective against chronic disease, nutrient deficiencies, including protein, are possible due to low bioavailability from plant-based sources. The consequences of inadequate dietary protein include reduced lean body mass (LBM) and muscle weakness. This study examined relationships between protein intake, strength, and LBM in 37 underactive vegetarians and recorded the impact of protein supplementation (18 g/day mung bean protein) on these indices utilizing an eight-week, randomized, controlled, feeding trial. Both handgrip and knee flexor and extensor strength were measured at baseline and week eight. At baseline, LBM was significantly related to grams of protein consumed daily. LBM was also correlated to grip strength (*r* = 0.569, *p* < 0.001) and lower body strength (*r* = 0.763 to 0.784; *p* < 0.001). Twenty-five vegetarians completed the feeding trial, including 11 in the protein supplementation group (PRO) and 14 in the control group (CON). At the end of the trial, LBM and strength did not differ significantly between groups. However, the average percent change for grip, flexor, and extensor strength did differ between PRO and CON participants (+2.9 ± 7.2% and −2.6 ± 7.3% respectively, *p* = 0.05). Thus, there were strong associations between dietary protein, LBM, and strength in vegetarians and an indication that supplementary vegetarian protein increased strength in the absence of exercise and independent of LBM.

## 1. Introduction

Interest in plant-based diets has grown in recent years with a focus on their relation to health and sustainability outcomes [1,2,3]. Plant-based diets generally include vegetarianism, which omits all animal flesh (e.g., beef, pork, poultry, and fish), lacto-ovo vegetarianism, which excludes animal flesh but includes dairy and eggs, and veganism, which restricts all animal products [4]. A 2016 Harris Poll conducted by the Vegetarian Resource Group found that just over 5% of U.S. adults aged 18–34 years self-identify as vegetarian, with at least half of these respondents identifying as vegan [5]. Recent acceptance and accessibility of these lifestyles have greatly increased with campaigns, such as Meatless Mondays, and an upsurge of plant-based protein food products in the marketplace [6,7,8].

Vegetarian and vegan diets have been found to be protective against cardiovascular disease, showing a 24% to 40% reduction in mortality versus omnivores [9,10,11]. This is largely attributed to reduced body mass index (BMI), low-density lipoprotein (LDL) and total cholesterol levels, and blood pressure in vegetarians and vegans versus their omnivore counterparts [12,13,14,15,16,17,18,19,20,21,22,23,24]. In addition, both lacto-ovo vegetarian and vegan diets are associated with a decreased incidence and risk for numerous types of cancers [25], and epidemiological evidence supports protection against metabolic disorders such as type 2 diabetes mellitus through decreases in fasting blood glucose and cholesterol levels in those following vegetarian diets [17,22,26,27,28,29,30,31,32].

Although the health benefits of vegetarian and vegan diets are well documented, concern remains over the potential of decreased nutrient intakes, which may be harmful in the long term [33]. While the Academy of Nutrition and Dietetics states that “…appropriately planned vegetarian diets, including vegan, are healthful, nutritionally adequate, and may provide health benefits for the prevention and treatment of certain diseases”, it is noted that several nutrients, including protein, should receive special attention when planning a meatless diet [34].

Current dietary reference intake (DRI) values for protein are 0.8 g/kg/day for the general population [35]. While the DRIs include vegetarian-specific recommendations for some nutrients such as iron, zinc, and calcium due to the lower bioavailability from plant-based sources, there is not a separate protein recommendation, even though plant protein can have digestibility scores that are 10%–30% lower than animal-based protein sources [36,37]. Additionally, low lean body mass (LBM) is consistently reported among vegetarians compared to their omnivore counterparts [38,39]. Consequently, others have suggested that vegetarians and vegans consume 1.0 g protein/kg/day with even higher levels recommended for exercisers and athletes [36,40].

Dietary protein acts not only as a building block in muscle protein synthesis (MPS), but the amino acid, leucine, acts as a stimulus in the signaling pathways for MPS [40,41]. During periods of inadequate protein intake, muscle protein breakdown can supersede MPS, causing atrophy and functional decline [42]. Muscle mass is a strong predictor of functional capacity, mobility, quality of life, and mortality [43,44], and reduced LBM is associated with loss of handgrip strength [45,46]. Studies have shown positive associations with higher protein intake and physical performance, specifically grip strength in older participants [47,48]. Grip strength is used as a functional marker of muscle mass and is one of the best predictors of stroke and myocardial infarction, as well as their recovery rates, and general disability later in life [49,50].

It is possible that the lower protein intake in vegetarians and vegans may relate to a decrease in grip strength [51]. Furthermore, there is limited research examining the effects of plant-based protein intake on strength and LBM independent of an exercise training component [38,52,53]. The present study was designed to examine relationships between strength, protein intake, and LBM in underactive vegetarian and vegan adults, as well as the impact of protein supplementation (18 g mung bean protein daily) on these indices. Both handgrip and knee flexor and extensor strength were measured in participants. A direct correlation between protein intake and strength was predicted; however, it was hypothesized that there would be no differences in strength, protein intake, and LBM between vegans and vegetarians.

## 2. Materials and Methods

Participants were recruited from the greater Phoenix, Arizona, area during August to October 2018 via fliers, word of mouth, email listservs, and local vegan Facebook groups. Inclusion criteria specified individuals 18–55 years old, vegetarian or vegan for at least one year, and healthy by self-report. Exclusion criteria included supplement use, such as protein powder or creatine; previous diagnosis of heart disease, cancer, stroke, diabetes, autoimmune disorders, or thyroid condition; competition in any athletic event in the past year; and moderate to strenuous exercise exceeding 150 minutes per week. Additionally, individuals were excluded if they were pregnant or planning to become pregnant. All participants completed the Physical Activity Readiness Questionnaire (PAR-Q) and were cleared for physical exertion.

A total of 343 individuals completed the online recruitment survey, and 124 of these individuals (36%) met the study criteria. Thirty-seven qualifying adults accepted the invitation to participate and provided written informed consent. The study was conducted in accordance with the Declaration of Helsinki, and the protocol was approved by the Arizona State University Institutional Review Board (STUDY00005383). The study is registered at ClinicalTrials.gov (identifier: NCT04076982).

Baseline measurements were completed in a single visit. Participants reported to the test site in a rested (no moderate to strenuous physical activity for 24 h) and fasted (no food or beverage with the exception of water for 8 h) state. Participants completed questionnaires covering demographics and a short health history. Height was recorded using a wall-mounted stadiometer, and body weight was measured using a calibrated scale (model TBF-300A, Tanita Corporation, Tokyo, Japan). Waist measurements were taken using a flexible tension tape at the minimal circumference. Physical activity was measured via a physical activity recall questionnaire and recorded in metabolic equivalent of task (METS) [54]. A 24 h dietary recall was administered by a trained researcher to assess the previous day’s intake, and the Food Processor software (ESHA Research, Salem, OR, USA) was used for all diet analyses using standardized default methods. LBM was measured via dual energy X-ray absorptiometry (DEXA) (Ge Lunar iDXA, Chicago, IL, USA) and was conducted by a trained X-ray technician. Dominant handgrip strength was measured in triplicate in a seated position with the elbow flexed to 90 degrees and a neutral wrist position using a handheld dynamometer (Takei Scientific Instruments, Niigata-City, Japan). An average of 3 consecutive measures was used for analyses. Lower body strength was measured in the dominant leg using a multi-joint system dynamometer (Biodex, Shirley, NY, USA). Isokinetic knee flexion and extension were measured from a seated position at a resistance of 90°/s. The participant was instructed to extend and flex their lower leg for 3 repetitions at a maximal effort. They performed 2 sets with a 30 second rest between sets. Next, isometric knee extension at a fixed 60° knee angle was performed. Participants were instructed to isometrically extend their lower leg against the arm of the dynamometer at a maximal effort for 5 seconds. They performed 3 repetitions separated by a 60 second rest between repetitions. Both peak and mean torque were recorded; the average mean torque was used for analyses.

Following baseline testing, participants were randomized to a protein supplementation group (PRO) (*n* = 19), which received the protein supplement (an egg-replacement patty manufactured from mung bean, JUST Egg, San Francisco, CA, USA; 18 g protein/day) or to a control (CON) group (*n* = 18), which received a control biscuit (belVita Breakfast Biscuit, Mondelez International, East Hanover NJ; 4 g protein/day). This level of supplemental protein (+18 g daily) surpasses the 12 g additional protein recommended in the published literature for vegetarian adults to supplement the RDA [36]. The test foods were matched for energy (200 kcal/day). Participants were instructed to consume the test foods in the morning hours and to keep a record of the days the foods were consumed on a study calendar, which was returned to investigators at the final visit and used to track protocol adherence. Participants were instructed to maintain all current exercise and dietary habits and not start any new medications during the trial period. At the completion of the intervention (trial week 8), all measurements conducted at baseline were repeated, including anthropometrics and DEXA scan, physical activity and diet assessment, and all strength measures. Twenty-five participants completed the trial in its entirety (11 and 14 in the PRO and CON groups, respectively). Twelve participants withdrew from the study for various reasons, including scheduling conflicts, acceptability of product, or unspecified reasons.

### Statistical Analysis

Power analyses indicated that 34 participants were needed to demonstrate a significant change in grip strength between groups (80% power; effect size = 2; SD = 2.0) [55]. Data are reported as the mean ± SD, an a priori α of 0.05 was used. Statistical analyses were performed using SPSS version 24 (IBM, Armonk, NY, USA). At baseline, Spearman’s rho correlation was used to examine relationships between variables, and an independent samples Mann-Whitney U test was conducted to compare mean differences in grip strength, protein intake, and LBM by diet group. At the completion of the 8-week trial, the change in the outcome data were normally distributed, and repeated measures ANOVA was utilized to identify significant changes between groups and effect size (Partial Eta Squared).

## 3. Results

Three men and 34 women were enrolled in the trial (12 vegetarians and 25 vegans). Demographic characteristics did not differ by diet type (Table 1). At baseline LBM was significantly related to grams of protein consumed daily (*r* = 0.340, *p* = 0.039). LBM was also correlated to average grip strength (*r* = 0.569, *p* < 0.001) and lower body strength (knee flexor and extensor muscles at 90°/s; *r* = 0.763 and 0.784 respectively; *p* < 0.001). These relationships remained significant when body weight and gender were controlled. Twenty-five participants completed the eight-week feeding trial (11 PRO and 14 CON; one male completed per group). Based on 24 h recalls at baseline and week eight, protein intakes (excluding the supplemental protein foods) did not vary over time or between groups: 46.1 ± 23.6 and 44.3 ± 26.7 g/day for PRO and 50.1 ± 17.3 and 54.6 ± 27.7 g/day for CON at baseline and week eight, respectively (*p* = 0.639, repeated measures ANOVA).

Physical activity did not differ between groups at baseline (27.0 ± 16.7 and 32.5 ± 20.3 MET hours/week for the PRO and CON groups respectively; *p* = 0.317) and did not change over time or between groups during the trial. Among all participants, mean body weight did not change over time, and weight change did not vary between groups during the study (−0.5 ± 2.1 and 0.0 ± 1.3 kg for PRO and CON, respectively, *p* = 0.495). Protocol adherence (days supplement consumed) did not vary significantly between groups (85% versus 91% for PRO and CON, respectively, *p* = 0.177). Outcome measures (change in LBM and change in strength) did not differ significantly between the study groups (Table 2). However, the average percent change in strength for grip, flexor, and extensor strength did differ significantly between PRO and CON participants (+2.9 ± 7.2% and −2.6 ± 7.3%, respectively, *p* = 0.05) when body mass and study adherence were controlled.

**Figure 1:**
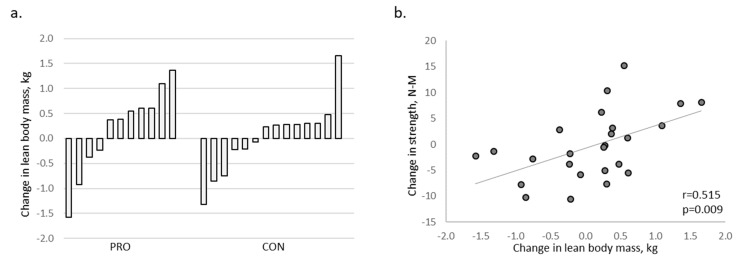
(**a**) Change in lean body mass by individual participant; 45% of PRO participants (18 g supplemental protein/day) gained >0.5 kg lean body mass during the trial versus 7% of CON participants (4 g supplemental protein/day). (**b**) Change in lean body mass in relation to change in knee flexor strength.

Overall, 45% of PRO participants gained >0.5 kg lean body mass during the eight-week trial versus 7% of CON participants (Figure 1a), and the change in lean body mass was correlated to knee flexor strength among all participants (*r* = 0.515; *p* = 0.009; Figure 1b).

## 4. Discussion

There were two main findings in this trial, a strong positive association between LBM and strength (both grip strength and knee flexor and extensor strength) and an indication that supplementary protein promotes strength without the adoption of a regimented exercise program and independent of LBM. The former observation was expected as greater muscle mass allows for greater force production capabilities [56]. However, the link between supplemental protein and greater strength, in the absence of exercise, is a novel finding. A number of studies already exist documenting gains in strength when exercise programs are paired with dietary protein supplementation; these studies generally have been oriented towards enhancing exercise performance [57,58,59]. The present study demonstrated for the first time that a vegetarian protein source alone and in the absence of exercise could contribute to strength outcomes in underactive plant-based eaters.

In the vegetarian participants, dietary protein intake (g/day), LBM, and body strength were directly related. These associations were expected, as dietary protein provides amino acid building blocks for muscle accretion, and acts as stimuli of physiologic pathways leading to MPS [52]. The mean protein intake of our sample was 0.76 g/kg/day, just slightly below the U.S. RDA (0.8 g/kg/day); yet, given the lower protein digestibility of vegetarian diets compared to omnivore diets, it has been suggested that vegetarians may require higher-than-RDA intakes, perhaps around 1.0 g protein/kg/day [36]. Thus, it is possible our participants were consuming considerably less than their actual protein needs. Further, the mean grip strength of participants at baseline (25.9 ± 7.3 kg) was significantly below the reference value for North American females (31 kg; *p* < 0.001) [60], which may be indicative of suboptimal protein intake in this population.

This pilot study was the first known investigation of the effect of a vegetarian protein supplement, mung bean protein isolate, in vegans and vegetarians in the absence of a training intervention. Mung beans are 20%–31% protein with an essential amino acid profile comparable to that of soy (18%–22%) and the FAO/WHO (1973) reference values [61,62,63,64,65]. Mung beans are high in essential amino acids, notably leucine, lysine, and phenylalanine while containing inadequate amounts of threonine, tryptophan, and the sulfur-containing amino acids cysteine and methionine [66]. Digestibility and bioavailability of mung beans is not yet well understood, with reports of in-vitro protein digestibility scores ranging from as low as 52% to 88% [61,67]. However, a recent study in humans using dual tracer techniques to measure indispensable amino acid digestibility at the small intestine found that mung bean protein had a digestibility of 56.7%, which was increased by an additional 9.9% if the mung beans were dehulled. These values were compared to that of spirulina (85.2%) and chickpeas (56.6%) [68]. Rutherfurd et al. showed similar ileal amino acid digestibility scores in rats consuming corn-based breakfast cereal (67%) and wheat bran (74%), two sources considered to have poor digestibility, while cooked peas (92%), soy protein isolate (96%), and whey protein isolate (100%) yielded far greater digestibility scores [69]. The previously reported relatively poor digestibility of mung bean might partially explain why there was no significant change in LBM or strength measures in the intervention group in the present study, although the average strength ratings (the average percent change in strength over eight weeks) did move in opposite directions during the study (PRO: +3%; CON: −3%; *p* = 0.05).

While the overall protein digestibility scores are low, the leucine content of mung bean protein isolate (74 mg/g) is considered high when compared to FAO/WHO guidelines (66 mg/g) [62]. While this may be the case, the total leucine content of mung beans (7.5%) is similar to other common plant-based sources such as peas (7.8%) and soy (8.0%), yet is still below that of animal-based sources such as milk (10.9%) and whey (13.6%) [52,70]. When discussing MPS, leucine is of special importance due to its ability to stimulate the mammalian target of the rapamycin (mTOR) pathway and through this, activate mRNA translation of new proteins while inhibiting the breakdown of existing proteins [71]. Pennings et al. showed a greater plasma leucine concentration following the ingestion of 20 g of whey versus casein or casein hydrolysate protein. They found a strong positive correlation between peak plasma leucine and muscle fractional synthetic rate, thus suggesting that greater postprandial leucine concentration might possess greater anabolic properties [72]. Further, Churchward-Venne et al. found that supplementation with 25 g of whey containing 3 g leucine or 6.25 g of whey supplemented with leucine to contain 5 g of total leucine yielded greater MPS than lower leucine formulations [73]. Given the results of these studies, it is possible that supplementation with 18 g of mung bean protein, as in the present study, was not sufficient to provide enough leucine (estimated at 1.35 g per day) to stimulate mTOR, and thus MPS. van Vliet et al. reported that 38 g of soy protein was required to provide 3 g of leucine [52]. Since the total leucine content of mung beans is slightly below that of soy, a mung bean protein supplement ≥38 g would be necessary to reach the proposed threshold of 3 g of leucine to stimulate mTOR.

Previous studies demonstrated that vegetarians generally have lower LBM than omnivores [37,39]. This may present several different problems. The present study showed a positive association between LBM and grip strength and LBM and lower body strength, which comports with previous findings of low LBM yielding lower grip strength [45,46]. In fact, Tong et al. found that even after adjustment for height, LBM, and physical activity, both male and female vegetarians had lower grip strength than omnivores [51]. Similar findings have also been reported by Varte et al. [74], but not by others [75]. Other studies showed that sarcopenic older adults reported decreased physical activity and lower overall quality of life [43], while decreased strength was associated with increased risk of all-cause mortality, regardless of LBM [44]. Further, grip strength is used as a functional marker of muscle mass as well as a predictor of stroke, myocardial infarction, and general disability [76,77,78]. Continued surveillance of LBM and strength in all adults may be an important strategy for predicting health and longevity. Particular attention should be paid to vegetarians and vegans, as they have been shown to oftentimes exhibit sub-normal values in these measurements.

Mung bean protein was chosen for this intervention due to its high protein content and sustainability of production, as mung bean crops do not require nitrogen fertilizers and use little water to grow [79]. Additionally, they provide a dietary staple in developing countries and regions where animal proteins are unavailable or cost-prohibitive [79].

Some factors presented limitations in this study. For example, participants’ diet information was collected utilizing a 24 h dietary recall questionnaire. Recalls are subject to a participant’s memory and honesty regarding the previous day’s diet and, as such, may not be accurate. It is also possible that days on which data were collected were not indicative of their usual diet. Also, while mung beans contain adequate amounts of several essential amino acids, such as leucine, lysine, and phenylalanine, they are lower in threonine and methionine compared to animal-based protein sources, such as whey. Therefore, even if a stimulus for MPS such as leucine was present, unless participants were eating an overall diet with varied protein sources containing high enough levels of all essential amino acids, the necessary building blocks for MPS might not have been present. This might have contributed to our results in which strength improved without increases in LBM.

## 5. Conclusions

This trial demonstrated strong associations between dietary protein, LBM, and strength in a small sample of healthy, underactive vegetarians and vegans. Since greater muscle mass allows for greater force production capabilities and muscular strength, future research should focus on quantifying the appropriate protein intake for individuals consuming mainly plant proteins. This trial provided preliminary evidence that supplementary plant protein (representing approximately 30% additional protein based on the current recommendation of 0.8 g protein/kg/day) increased muscular strength in the absence of exercise and independent of LBM.

## Figures and Tables

**Table 1:** Participant characteristics *.

	Age (year)	Weight (kg)	BMI (kg/m^2^)	Lean Body Mass (kg)	Average Grip, kg	Protein (g/kg/ day)
Vegan (*n* = 25; 2/23 M/F)	31.3 ± 9.3	63.0 ± 13.5	23.4 ± 4.1	39.0 ± 8.0	26.8 ± 8.0	0.74 ± 0.30
Vegetarian (*n* = 12; 1/11 M/F)	31.0 ± 9.5	70.8 ± 19.2	25.2 ± 5.7	40.6 ± 9.1	24.0 ± 5.4	0.79 ± 0.29

* Values are Mean ± SD; no significant differences between diet groups (*p* > 0.2).

**Table 2:** Change in lean body mass and strength in PRO and CON participants *.

	PRO (*n* = 11; 1/10 M/F)			CON (*n* = 14; 1/13 M/F)			*p*	Effect
	Pre	Post	∆	Pre	Post	∆	Value	Size
Lean body mass, kg	39.7 ± 8.3	39.3 ± 8.8	+0.2 ± 0.9	39.9 ± 8.3	39.4 ± 8.7	0.0 ± 0.7	0.598	0.012
Average grip, kg	24.2 ± 5.5	24.7 ± 4.5	+0.5 ± 2.1	26.3 ± 9.5	26.2 ± 9.4	−0.1 ± 1.6	0.409	0.030
Knee flexor (90°/s), N-M	72.4 ± 19.9	74.0 ± 18.4	+1.5 ± 6.4	72.6 ± 24.9	70.8 ± 23.7	−1.8 ± 6.4	0.211	0.067
Knee extensor (90°/s), N-M	96.8 ± 30.0	98.0 ± 33.7	+1.2 ± 14.1	96.6 ± 38.6	90.1 ± 36.3	−6.5 ± 12.1	0.153	0.087

* Values are Mean ± SD; no significant differences between groups at baseline (*p* > 0.2). PRO (experimental group, 18 g supplemental protein/d); CON (control group, 4 g supplemental protein/d).
